# Tibial base design and patient morphology affecting tibial coverage and rotational alignment after total knee arthroplasty

**DOI:** 10.1007/s00167-014-3402-x

**Published:** 2014-10-31

**Authors:** Chadd Clary, Luke Aram, Daren Deffenbaugh, Mark Heldreth

**Affiliations:** DePuy Synthes Joint Reconstruction, 46582 Warsaw, IN USA

**Keywords:** Total knee arthroplasty, Tibial coverage, Tibial anthropometrics, Surgical technique

## Abstract

**Purpose:**

To understand interactions between total knee arthroplasty tibial base design attributes, variations in tibial morphology, and the resulting tibial coverage and rotational alignment.

**Methods:**

Tibial anthropometric measurements, including aspect ratio (medial–lateral width/anterior–posterior length) and tibial asymmetry, were taken for 14,791 total knee arthroplasty patients and compared with the ability of four different commercial tibial base designs to cover the resected plateau. The anthropometric measurements were also compared with the resulting tibial base rotation, which occurred when rotating the base to maximize coverage.

**Results:**

All four tibial base designs resulted in similar coverage ranging from 80.2 (4.7) % to 83.8 (4.6) %. Mean tibial base rotation when placed to maximize coverage ranged from 3.7 (4.4)° (internal) to 3.8 (4.5)° (external) relative to the medial third of the tibial tubercle. More asymmetric tibiae and tibiae with a lower aspect ratios resulted in increased internal tibial base rotation.

**Conclusions:**

The four tibial base designs assessed provided similar levels of tibial bone coverage across the patient population, despite different design features. Rotating the tibial base to maximize coverage did not significantly increase the tibial coverage, but induced variability in tibial base alignment. Certain tibial anthropometrics may predispose particular patients to internal tibial base mal-rotation.

## Introduction

Optimizing tibial base rotational alignment [1, 11, 13] and maximizing tibial coverage [4, 9] are important factors to ensuring the long-term survivorship and function of total knee arthroplasty (TKA). Targets for rotational alignment of the tibial base are well documented [1, 11, 13], and tibial mal-rotation has been associated with increased rates of knee revision [2, 3, 20]. Despite frequent failures due to aseptic tibial loosening [7, 8, 15, 19, 22], a minimum level of tibial coverage to ensure long-term fixation has not been demonstrated. Furthermore, tibial base designs which increase the amount of tibial coverage have not been clinically associated with reduced rates of aseptic tibial loosening.

The surgeon’s task of choosing the optimum tibial base geometry and alignment is complicated by the large variations in tibial morphology across the patient population. Patients with increased asymmetry of the tibial plateau (longer medial plateau than lateral plateau) may lead the surgeon to internally mal-rotate the tibial base to attain a more desirable coverage [18]. Conversely, asymmetric tibial bases may necessitate external mal-rotation in patients with a relatively symmetric tibial plateau. Ideally, tibial base design attributes, including the peripheral shape of the tibial base, asymmetry between the medial and lateral plateaus, and the number and distribution of tibial base sizes, should ensure robust coverage across the patient population while enabling proper rotation [25]. To optimize this function, some authors advocate the use of asymmetric tibial bases to mimic the asymmetry of the native tibia [10, 18], while others advocate rotating platform (RP) TKA. Theoretically, RP tibial bases decouple the rotation of the tibial insert from the tibial base, allowing the base to be placed to maximize coverage without adversely affecting knee mechanics [14, 21].

**Fig. 1:**
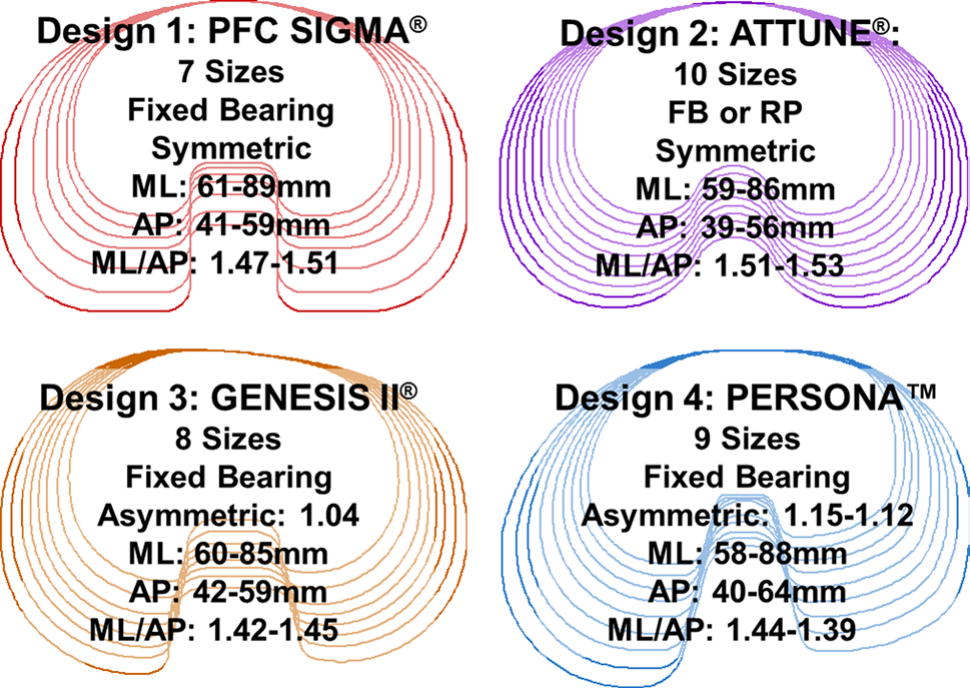
The four tibial base systems, their associated sizing distribution, and geometric measurements

The purpose of the current study was to understand interactions between tibial base design attributes, variations in tibial morphology, and the resulting tibial coverage and tibial base alignment. Understanding these interactions would guide surgeons when choosing the optimum tibial base design for a particular patient’s morphology, improving tibial coverage and reducing the risk of tibial mal-rotation. To this end, the tibial coverage of four modern tibial base designs with varying design attributes (Fig. 1) was assessed across a large patient population. In addition, the basic morphology of the tibiae was measured, and the effect of tibial morphology on tibial base coverage and alignment was quantified. The hypothesis of the current study was that increasing the number of tibial base sizes would improve tibial coverage independent of tibial morphology, while tibial asymmetry would improve coverage only in patients with more asymmetric tibiae.

## Materials and methods

Lower limb computed tomography scans were collected from 14,791 subjects with end-stage osteoarthritis. The subjects had a mean height of 1.68 (0.11) m and weight of 85 (15) kg. The preoperative hip–knee angle frontal plane alignment of the subjects was 2.9 (5.4)° varus. The population was 62 % female, and 53 % were right knees. 85.2 % of the patients were from North America, while 10.6 % were from Europe, 2.3 % from Asia, 1.4 % from Australia, and the remaining 0.5 % from the Middle East and Africa.

**Fig. 2:**
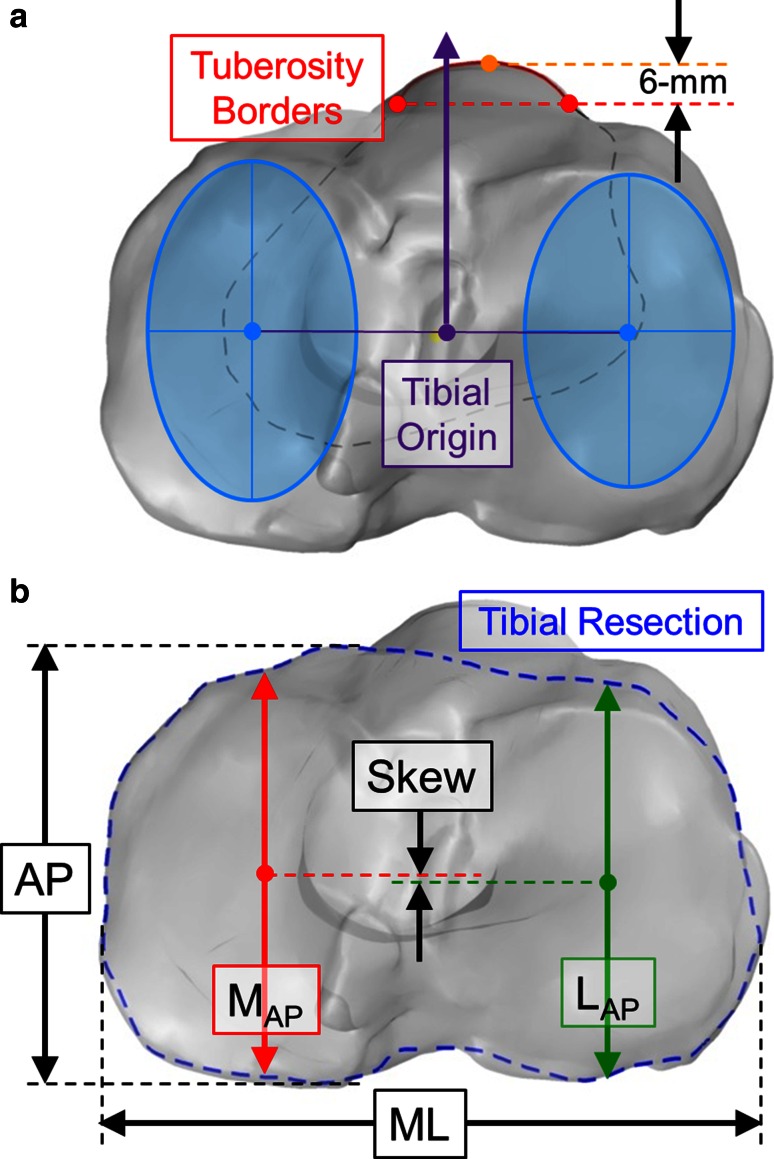
Sample anthropometric measurements taken for each subject in the study. The midpoint between the geometric centers of the medial and lateral plateaus was used to define the tibial origin, and the A–P axis was aligned to the medial third of the tubercle (**a**). The overall A–P and M–L dimensions of the resected plateau were measured, in addition to the A–P lengths of the medial and lateral plateaus and the A–P offset between the midpoints of the medial and lateral plateau (**b**)

For each subject, the tibial bone was manually segmented and anatomic landmarks were identified to establish the tibial coordinate system. The tibial superior–inferior (S–I) axis was established along a line originating from the midpoint between the centers of the medial and lateral tibial plateaus (tibial origin) and terminating at the midpoint between the medial and lateral malleoli. The tibiae were oriented along their S–I axis and preliminarily rotated transversely to a medial–lateral (M–L) axis, connecting the centers of the medial and lateral tibial plateaus. From this orientation, the most anterior point on the tibial cortex was identified as the tibial tuberosity. A cross section perpendicular to the tibial S–I axis was extracted through the apex of the tuberosity, and the medial and lateral borders of the tuberosity were defined at the intersections of the cross section and a M–L line offset 6 mm posterior to the apex of the tubercle (Fig. 2a). From these points, the overall width of the tibia tubercle was calculated and the medial third of the tuberosity was identified. The tibia was then rotated about the S–I axis such that the anterior–posterior (A–P) axis of the tibial coordinate system was parallel to a vector connecting the origin of the tibia to the medial third of the tibial tubercle.

In this orientation, a virtual surgery was performed with an 8-mm tibial resection (referencing the high side of the tibia plateau along the S–I axis) made perpendicular to the tibial S–I axis in the frontal plane with 3° of posterior slope in the sagittal plane. The intersection of the resection plane and the outer cortex of the tibial bone were extracted for each subject. A series of anthropometric measurements were taken to quantify the morphology of the tibial resection (Fig. 2b). The overall M–L and A–P dimensions of the tibial resection were extracted, and the aspect ratio of the resection was calculated (M–L/A–P). To characterize the asymmetry of the resection, the overall A–P length of the medial and lateral plateaus was extracted along a sagittal cross section midway between the tibia origin and the medial and lateral tibial borders. The asymmetry of the resection was defined as the ratio of the medial A–P plateau length by the lateral A–P plateau length. An additional measure of asymmetry, tibial skew, was calculated by the A–P offset between the midpoints of the medial and lateral A–P cross sections (with anterior offset of the lateral plateau relative to the medial plateau as positive).

An automated algorithm was used to optimize the size and placement of the tibial base on the resected plateau by minimizing the amount of tibial base overhanging the outer profile of the tibial resection for each size of tibial base in the knee system. Each size tibial base was initially centered on the mid-plane of the tibial resection along the M–L axis, with the anterior border of the base adjacent to the anterior aspect of the tibial resection, and rotationally aligned to the medial third of the tibial tubercle. From this position, the M–L and A–P position of the tibial base was optimized using an unconstrained nonlinear optimization algorithm in Matlab™ (Mathworks, Natick, MA, USA).

The optimization function was the summation of the base overhang at 320 points placed uniformly around the periphery of the tibial base (160 points on both the medial and lateral plateaus). Points in the intercondylar notch of the base were excluded from the cost function as bone is frequently preserved in this region to protect the tibial attachment of the posterior cruciate ligament. The amount of overhang at each point was calculated by finding the distance from the tibial base profile to the resected tibia profile along a vector normal to the base’s outer profile. A value of zero was assigned if the point on the tibial base was within the profile of the resected tibia. Otherwise, the magnitude of the base overhang was added to the optimization function. The largest sized tibial base in the system that could be positioned with <2 mm of base overhang at any point around the periphery of the resected plateau was selected as the patient’s optimized base size and position when aligned to the medial third of the tibial tubercle. Tibial base overhang of up to 2 mm was allowed because previous clinical studies have shown that <3 mm of implant overhang was not associated with compromised outcomes after knee arthroplasty [5, 17]. The optimization was then repeated using a “maximum coverage” philosophy, including the tibial base internal–external (I–E) rotation in the optimization algorithm to simulate the surgical practice of maximizing tibial coverage without regard for rotational alignment, as may be done with a RP-TKA. To quantify the resulting tibial coverage, the surface area of the tibial base was divided by the area of the resected plateau.

The analysis was performed with four different tibial base systems composed of varying size offerings, peripheral shapes, and levels of tibial asymmetry (Fig. 1). The percentage of tibial coverage and the base under-hang at each point on the base profile were averaged across the patient population for each base type and placement philosophy (medial third or maximum coverage). To quantify the influence of tibial morphology on tibial base function, the subject population was divided into subgroups based on each metric of tibial anatomy (aspect ratio, asymmetry, skew). The tibial coverage (when aligned to the medial third of the tubercle) and rotational alignment (when placed to maximum coverage) for each tibial base system was averaged across each subgroup. Paired *t* tests were conducted to assess significant differences between the coverage attained by the various tibial base designs. Due to the large subject population, traditional statistical methods demonstrated that all differences between coverage and overhang across the tibial base designs were statistically significant, even if not clinically significant. Therefore, only mean and standard deviations have been reported.

## Results

**Fig. 3:**
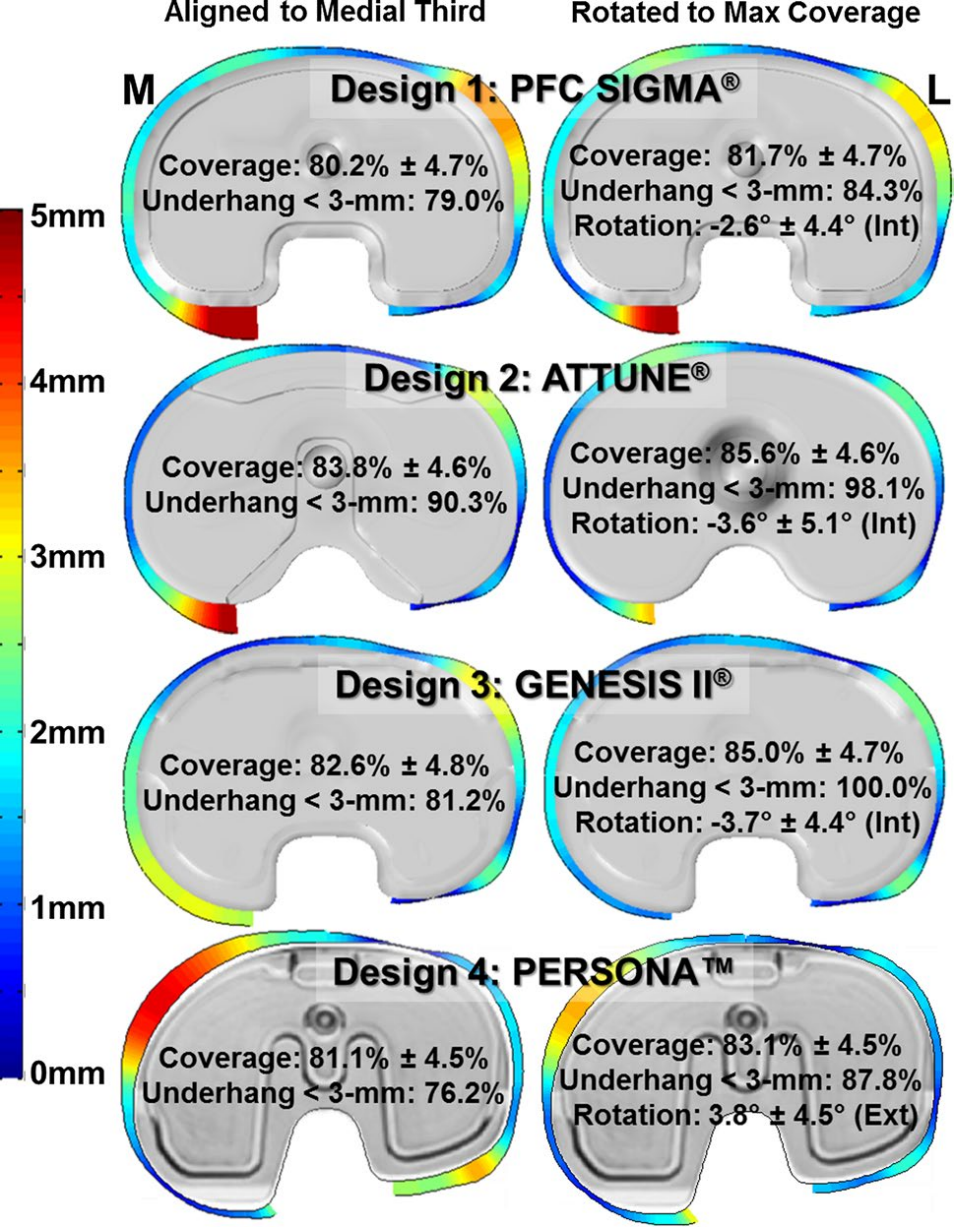
The overall coverage, the percentage of the tibial periphery with greater than 3 mm of exposed bone, and the magnitude of exposed around the implant periphery of the various tibial base designs when aligned to the medial third of the tibial tubercle (*left column*) or rotated to maximize coverage (*right column*)

When aligned to the medial third of the tibia tubercle, all tibial base systems had similar levels of overall coverage across the patient population, ranging from 80.2 (4.7) % (Design 1) to 83.8 (4.6) % (Design 2), despite their design differences (Fig. 3). While not evident in the overall coverage, the influence of the design factors could be seen on the location and magnitude of the exposed bone around the base periphery. The outer periphery of the tibial base (excluding the PCL notch) was within 3 mm of the resected periphery along 79.0 % of the periphery length for Design 1, 90.3 % for Design 2, 81.2 % for Design 3, and 76.2 % for Design 4. The symmetric designs had >3 mm of exposed bone along the poster medial cortex (Designs 1 and 2) and along the anterior lateral cortex (Design 1). The asymmetric designs had different patterns of exposed bone, with >3 mm of exposed bone on the poster medial and anterior lateral cortex for Design 3 and along the anterior medial and posterior lateral cortex for Design 4.

When rotated to maximize coverage (Fig. 3), the increase in overall coverage ranged from 1.5 % (Design 1) to 2.4 % (Design 3). The pattern of exposed bone was similar in both alignment conditions. When rotated to maximize coverage, the exposed bone posterior medially for Designs 1–3 was reduced, while the exposed bone anterior medially and posterior laterally was reduced for Design 4. Rotating the base to maximize coverage had a large effect on the tibial base alignment. On average, Designs 1–3 rotated internally relative to the medial third of the tibial tubercle between 2.6 (4.4)° (Design 1) and 3.7 (4.4)° (Design 3). Conversely, Design 4 rotated externally relative to the tubercle by a mean of 3.8 (4.5)°.

**Fig. 4:**
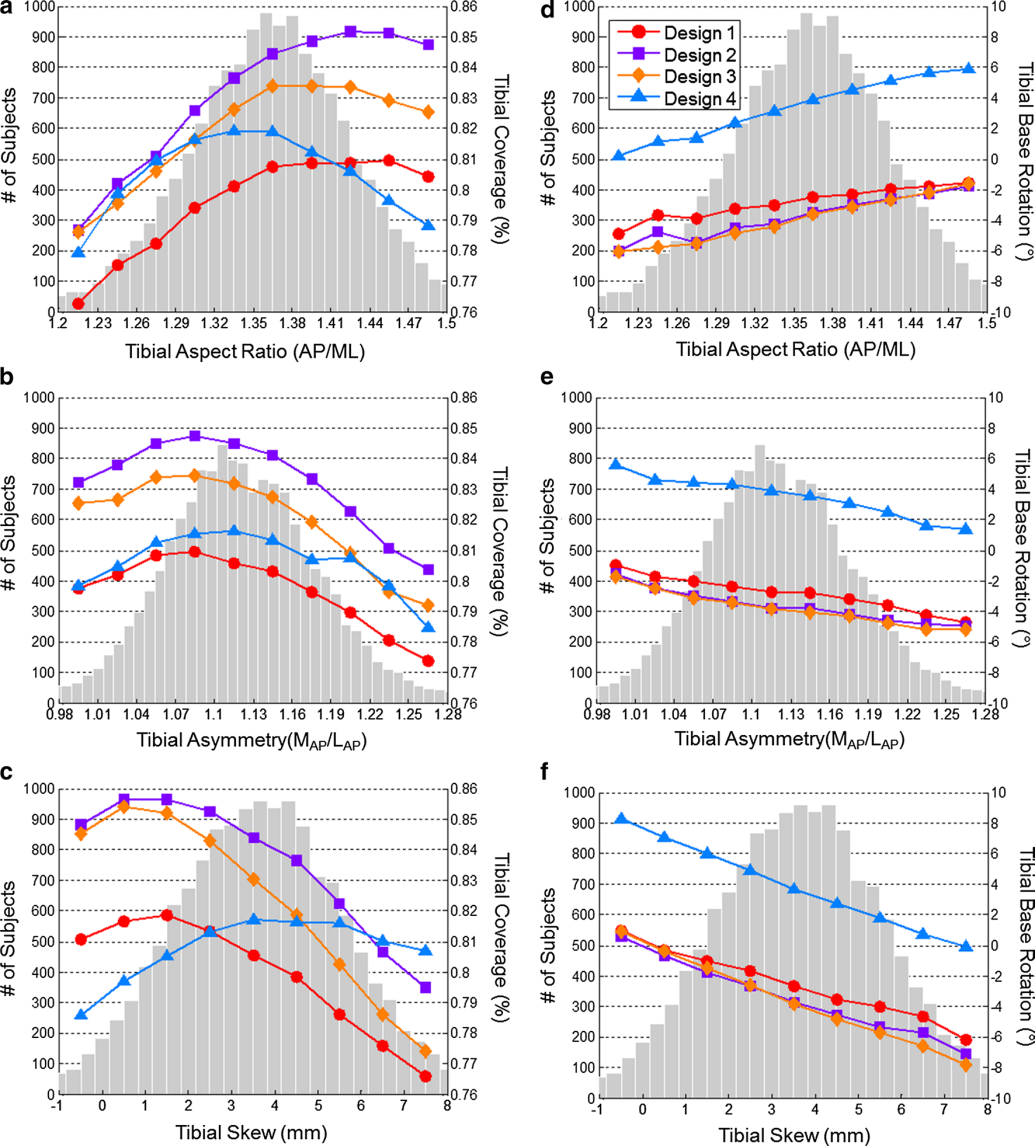
The mean tibial coverage when aligned to the medial third of the tubercle (*left column*) and tibial base rotation when maximizing coverage (*right column*) for the four tibial base designs (shown on the *right axis*) as a function of tibial aspect ratio (**a**, **d**), tibial asymmetry (**b**, **e**), and tibial skew (**c**, **f**). Coverage and rotation are superimposed over the histogram for each tibial morphology measurement across the subject population (*left axis*)

Variations in tibial morphology had similar effects on tibial coverage for Designs 1–3, but affected Design 4 differently (Fig. 4). Designs 1–3, which had larger aspect ratios than design 4, provided their peak coverage for patients with aspect ratios above the mean aspect ratio of 1.36 (0.07). Conversely, Design 4 provided its best coverage for subjects with an aspect ratio that was smaller than the mean. All bases provided the worst coverage for tibiae with the smallest aspect ratios. All four tibial base designs provided their best coverage for subjects with asymmetry near, or slightly less than the mean tibial asymmetry of 1.12 (0.10). Subjects with the highest levels of tibial asymmetry had the worst fit for all tibial base designs. Designs 1–3 provided their peak coverage for patients that had minimal tibial skew (the lateral plateau between 0 and 2 mm anterior of the medial plateau). Conversely, Design 4 provided its best coverage for patients with tibial skew between 3 and 6 mm.

When rotated to maximize coverage, tibial morphology had a consistent influence on the relative rotation of the tibial base to the tibial tubercle for all implant designs (Fig. 4). Tibiae with higher levels of tibial skew (more anterior lateral plateaus), lower aspect ratios (narrow tibiae), and higher levels of tibial asymmetry (longer medial plateaus) resulted in more internal rotation of the tibial base relative to the medial third of the tubercle. Independent of tibial morphology, Design 4 was consistently rotated 6–8° external to Designs 1–3.

## Discussion

The most important finding of the current study was that all tibial base designs evaluated provided a reliable level of tibial coverage, between 80.2 and 83.8 %, independent of the base design attributes. While several studies have assessed the tibial coverage of various TKA systems [6, 12, 16, 18, 23–25], only two studies utilized similar rotational alignment and sizing philosophies that enabled detailed comparisons [24, 25]. Wernecke et al. reported tibial coverage of six tibial base designs across a population of 101 young, healthy subjects ranging from 80 to 88 % [24]. Two of the designs assessed by Wernecke et al. were also assessed in the current study (Design 1 and Design 3). In both instances, the reported coverage by Wernecke et al. was 5 % higher than the current study. These differences could be attributed to the method of implant size selection, where Wernecke et al. were more tolerant of base overhang, which would allow placement of a larger-size tibial base than in the current study. Westerich et al. [25] assessed the fit of three historic base designs, two of which were predecessors to Designs 1 and 3 in the current study, across 42 TKA patients. They found the mean coverage of these trays ranged between 83.68 and 86.24 %, slightly higher than the current study.

Due to the relatively similar levels of tibial coverage across designs, it was unclear which design features led to the most reliable coverage. Design 2, which had the highest overall coverage of the tibial bases analyzed, was symmetric, had the highest aspect ratio, and had the most sizes confirming the hypothesis that increased sizing options robustly provided the best fit independent of patient anatomy. Tibial base asymmetry influenced the fit of Designs 3 and 4 differently. The proud anterior medial aspect and longer posterior medial plateau of Design 3 reduced the amount of exposed bone in these regions compared with the symmetric design. In Design 4, however, the tibial asymmetry left more bone exposed anterior medially. The two asymmetric base designs also interacted differently with the amount of tibial asymmetry and skew disproving the hypothesis that asymmetric base designs would provide better coverage for more asymmetric tibiae. Design 3 provided the best coverage for patients with lower levels of tibial skew where Design 4 provided the best coverage with high levels of tibial skew, indicating that the shape of the base asymmetry plays a critical role in the achievable tibial coverage. In general, it is unclear if these small differences in the amount of exposed bone have any significant influence on implant longevity as clinical studies have not associated tibial base design features with improved tibial fixation. However, assessments of bone quality at the tibial resection indicate weaker bone along the anterior cortex, which predisposes tibial bases to anterior subsidence [4]; therefore, coverage in this region may be particularly important to prevent loosening.

The practice of rotating the tibial base to maximize tibial coverage did not result in a significant increase in tibial coverage and led to large variations in base alignment. For Designs 1–3, this practice led to internal rotation of the tibial base by a mean of 3–4°, while in Design 4, maximizing coverage caused external rotation of the base by almost 4°. For all base designs, the amount of rotation imparted was highly variable with standard deviations between 4.4° and 5.1°. None of these tibial base designs (symmetric or asymmetric) were reliably aligned to the medial third of the tubercle with the coverage maximized across the patient population. In a comparable finding, Martin et al. [18] found that when maximizing coverage, both symmetric and asymmetric tibial base designs resulted in >5° of internal mal-rotation of the tibial base for between 28 and 100 % of the subjects depending on the implant asymmetry. The mean internal rotation found by Martin et al. was between 2 (5)° and 14 (5)° depending on the tibial base design, which was more internal than reported in the current findings. Martin et al. used a sizing algorithm that did not allow overhang of the tibial base, which would have required smaller-sized trays than the current study, enabling more internal rotation when maximizing coverage. While it is unclear based on the current analysis whether the mal-rotation caused by maximizing tibial coverage would lead to clinical complications, the data do indicate that if clinicians choose to set their rotation based on maximizing coverage, they should be aware of the interaction between tibial anthropometrics and the rotational bias created by the design attributes of a particular tibial base. Both Martin et al. and the current study conclude that setting rotational alignment by maximizing coverage should be avoided for all base designs, except potentially for RP tibial bases where the insert is not rotationally coupled to the base.

The current study is unique in that the subject population was very large and all subjects were potential candidates for TKA. The subject population used in this analysis was predominately North American and European, so caution should be used when applying these findings to patients of different ethnicity. Analyzing such a large subject population presented some additional limitations. In particular, the sizing and placement of the tibial base were done through an automated optimization algorithm that provided reliable placement, but was unable to recognize some unique clinical challenges encountered intraoperatively. Surgical decisions such as increasing the resection depth or posterior slope to improve ligament balance would not be accounted for in the current tibial resection. The medial third of the tubercle was chosen as a reliable rotational landmark [13], although patient and surgical variability may lead to an alternate ideal alignment for a given patient. In addition, the algorithm was not capable of identifying the formation of osteophytes or significant bone defects that may influence the resulting tibial resection profile or placement of the tray. Finally, the current analysis utilized total coverage to quantify tibial base performance, but did not take into consideration the quality of bone that supported the tray. Future work will focus on understanding the influence of surgical technique on both the tibial coverage and on the tibial bone quality that supports the tibial base.

## Conclusion

In summary, the current study demonstrated that the four modern tibial base designs assessed provided similar levels of tibial bone coverage across a large patient population, despite different design features. The practice of rotating the tibial base to maximize coverage did not significantly increase the amount of tibial coverage, but did induce a high level of variability in tibial base alignment relative to the tibial tubercle, which may compromise knee mechanics. Surgeons should be particularly careful when rotating the tray to maximize coverage if the tibia has a lower aspect ratio and is highly asymmetric as this may increase the risk of excessive internal base rotation.
